# Differential methylation EPIC analysis discloses cisplatin-resistance related hypermethylation and tumor-specific heterogeneity within matched primary and metastatic testicular germ cell tumor patient tissue samples

**DOI:** 10.1186/s13148-021-01048-y

**Published:** 2021-04-06

**Authors:** João Lobo, Vera Constâncio, Pedro Leite-Silva, Rita Guimarães, Mariana Cantante, Isaac Braga, Joaquina Maurício, Leendert H. J. Looijenga, Rui Henrique, Carmen Jerónimo

**Affiliations:** 1 Cancer Biology and Epigenetics Group, IPO Porto Research Center (GEBC CI-IPOP), Portuguese Oncology Institute of Porto (IPO Porto) & Porto Comprehensive Cancer Center (P.CCC), R. Dr. António Bernardino de Almeida, 4200-072 Porto, Portugal; 2 Department of Pathology, Portuguese Oncology Institute of Porto (IPOP), R. Dr. António Bernardino de Almeida, 4200-072 Porto, Portugal; 3 Department of Pathology and Molecular Immunology, Institute of Biomedical Sciences Abel Salazar, University of Porto (ICBAS-UP), Rua Jorge Viterbo Ferreira 228, 4050-513 Porto, Portugal; 4 Princess Máxima Center for Pediatric Oncology, Heidelberglaan 25, 3584 CS Utrecht, The Netherlands; 5 Department of Urology, Portuguese Oncology Institute of Porto (IPOP), R. Dr. António Bernardino de Almeida, 4200-072 Porto, Portugal; 6 Department of Medical Oncology, Portuguese Oncology Institute of Porto (IPOP), R. Dr. António Bernardino de Almeida, 4200-072 Porto, Portugal

**Keywords:** Testicular germ cell tumors, Metastasis, Cisplatin, DNA methylation, EPIC array

## Abstract

Testicular germ cell tumors (TGCTs) are among the most common solid malignancies in young-adult men, and currently most mortality is due to metastatic disease and emergence of resistance to cisplatin. There is some evidence that increased methylation is one mechanism behind this resistance, stemming from individual studies, but approaches based on matched primary and metastatic patient samples are lacking. Herein, we provide an EPIC array-based study of matched primary and metastatic TGCT samples. Histology was the major determinant of overall methylation pattern, but some clustering of samples related to response to cisplatin was observed. Further differential analysis of patients with the same histological subtype (embryonal carcinoma) disclosed a remarkable increase in net methylation levels (at both promoter and CpG site level) in the patient with cisplatin-resistant disease and poor outcome compared to the patient with complete response to chemotherapy. This further confirms the recent results of another study performed on isogenic clones of sensitive and resistant TGCT cell lines. Differentially methylated promoters among groups of samples were mostly not shared, disclosing heterogeneity in patient tissue samples. Finally, gene ontology analysis of cisplatin-resistant samples indicated enrichment of differentially hypermethylated promoters on pathways related to regulation of immune microenvironment, and enrichment of differentially hypomethylated promoters on pathways related to DNA/chromatin binding and regulation. This data supports not only the use of hypomethylating agents for targeting cisplatin-resistant disease, but also their use in combination with immunotherapies and chromatin remodelers.

## Introduction

Germ cell tumors (GCTs) comprise a heterogeneous group of neoplasms that arise in both genders—in the gonads (testis and ovary) and also in extragonadal sites (related to migration of primordial germ cells along the midline of the body)—and within a wide age range, from pediatric age (type I) to adolescence/adulthood (type II) and older age (type III) [1]. GCTs are developmental cancers, as their pathobiology closely resemble germ cell and embryonic development, in a way that they recapitulate the specific epigenetic status of the respective cell of origin [2]. Of all seven distinct classes of GCTs, the type II tumors of the testis (TGCTs) are by far the most common and present most clinical challenges, including those related to early diagnosis, appropriate treatment strategies, adequate follow-up and emergence of metastatic disease and resistance to platin-based chemotherapy [3]).

DNA methylation is the most studied epigenetic mechanism overall, and specifically in cancer [4]. DNA methylation-based biomarkers are attractive for aiding in clinical decision, given improvements in methodologies for their accurate detection and quantification, including non-invasively (i.e. in liquid biopsies). Several gene promoters and specific panels have shown promise in early diagnosis/screening, but also as indicators of patient prognosis, namely for prediction of relapses, metastatic events and response to systemic treatments [5, 6]. Specifically, in TGCTs, distinct methylation patterns are recognized among the major histological subtypes, seminomas and non-seminomas [7–9], and also across the individual non-seminoma subclasses, as a reflection of a differentiation-coupled methylation reprogramming [10–12]. However, few studies have dedicated to exploring net changes in the methylome between primary TGCTs and respective metastases [13], and especially between cisplatin-sensitive and cisplatin-resistant tumors, despite evidence on isogenic sensitive/resistant cell line clones demonstrating the relevance of epigenetics in the emergence of such resistance [14–17]. In fact, global hypomethylation has been suggested to be in part responsible for the outstanding sensitivity to cisplatin. This lack of studies in this niche is in part because tissue samples from metastatic locations with remaining viable tumor are rarely available, with most studies focusing on the investigation of chemo-naïve primary tumor samples [18], which has limitations. Although recent studies have provided big data analyses on copy number variations and mutations [19–22], and an interesting recent study has provided strong and complete data on differential mRNA expression among sensitive and resistant cell lines [23], genome-wide methylation data in paired clinical samples is lacking.

In this work, we make use of a set of well-characterized TGCT samples, comprising matched primary and metastatic tumors with differential exposure to cisplatin-based chemotherapy, and perform 850 k EPIC methylation array for analyzing differential methylation changes between patient samples.

## Methods

### Clinical samples

A total of twelve type II TGCT individual samples, belonging to four patients, were prepared for EPIC methylation array and included in the study: patient #1 with a primary testicular mixed tumor (for which two individual components, yolk sac tumor and teratoma were individually dissected) and a yolk sac tumor bone metastasis; patient #2 with a primary testicular seminoma and a seminoma lymph-node metastasis; patient #3 with a primary testicular embryonal carcinoma and four chemo-exposed metastases with viable embryonal carcinoma, two in the lung and two in lymph-nodes (the patient showed progressive cisplatin-resistant disease and died of disease); and patient #4 also with a primary testicular embryonal carcinoma and an embryonal carcinoma lung metastasis, who showed a complete response to cisplatin-based chemotherapy. Detailed clinicopathological information about the samples/patients is provided in Table 1. All patients were diagnosed and treated by the same multidisciplinary team at the Portuguese Oncology Institute of Porto, Portugal. Specimens were formalin-fixed and paraffin-embedded, and 10 µm sections were ordered from a representative block for DNA extraction. All samples had > 80% tumor cellularity and were further macro-dissected to eliminate foci of necrosis and hemorrhage. All tissue samples were reviewed by the same TGCT-dedicated Pathologist, according to the most recent World Health Organization (WHO) 2016 Classification, as previously reported by us [24]. Clinical charts were also reviewed and patients staged according to the most recent American Joint Committee on Cancer (AJCC) 8th Edition [24]. This study was approved by the Ethics Committee of IPO Porto (CES-IPO-12-018).

**Table 1:** Clinicopathological characterization of the samples included in the work

Sample	Patient	Age at diagnosis	Histology	Primary versus metastasis	Topography	Stage at diagnosis	Treatment course
16YST	Patient #1	29	Mixed tumor, YST component	Primary	Left testis	III (pT2N3M1b)	Orchiectomy + emergent laminectomy → 8 courses CT (4BEP + 4VeIP) + RT (bone) Complete remission, serious toxicity and paresis ANED
16TE			Mixed tumor, TE component		Left testis		
M6YST			YST	Metastasis	Bone		
117SE	Patient #2	33	SE	Primary	Right testis	II (pT1aN2M0)	Inguinal LN excision → orchiectomy → RT Complete remission ANED
M12SE			SE	Metastasis	Inguinal LN		
255EC	Patient #3	21	EC	Primary	Right testis	II (pT2N2M0)	Orchiectomy → 3 courses CT (3BEP) → disease progression under CT (lung biopsy: viable EC) → 9 courses CT (4VeIP + 4TIP + 1GEMOX) → LND and lung resection (viable disease, M20EC and M21EC) → disease progression → second LND and lung resection (viable disease, M22EC and M23EC) Cisplatin resistance, disease progression DoD
M20EC			EC	Metastasis	Lung		
M21EC			EC	Metastasis	Mediastinal LN		
M22EC			EC	Metastasis	Lung		
M23EC			EC	Metastasis	Mediastinal LN		
27EC	Patient #4	22	EC	Primary	Left testis	III (pT1N3M1a)	Lung biopsy → orchiectomy → 4 courses CT (4BEP) → LND (no viable tumor) Complete remission ANED
M4EC			EC	Metastasis	Lung		

*ANED* alive with no evidence of disease, *CT* chemotherapy, *DoD* dead of disease, *EC* embryonal carcinoma, *LN* lymph node, *LND* lymph node dissection, *SE* seminoma, *TE* teratoma, *YST* yolk sac tumor

### EPIC methylation array

DNA was extracted using the RNA/DNA Purification Plus Kit (Norgen, Canada, USA) and bisulfite-treated using the EZ DNA Methylation™ Kit (Zymo Research), according to the correspondent manufacturers’ instructions. Then, it was subjected to Illumina EPIC BeadChip (Illumina, San Diego, USA). DNA methylation data from the EPIC bead array was analyzed using the RnBeads package version 2.6.0 [25] for R 4.0.2, including import, quality control, SWAN normalization, and exploratory and differential analysis (including GO enrichment analysis related to molecular function). All CpG positions were mapped against the human hg19 reference genome.

### Data analysis

Unsupervised hierarchical clustering was performed using correlation-based dissimilarity metric and complete linkage.

Differential methylation (both regional-level—promoters—and site-level) was computed based on a combined rank, that included for each site the difference in mean methylation levels of the two groups being compared, the quotient in mean methylation and p-values computed using the limma method. The top 1000 combined ranks were considered as differentially hyper/hypomethylated for all downstream analyses.

Venn diagrams were assembled to evaluate the number of hyper/hypo-methylated gene promoters shared across histological types and between the different patients.

## Results

### Exploratory analyses

**Fig. 1:**
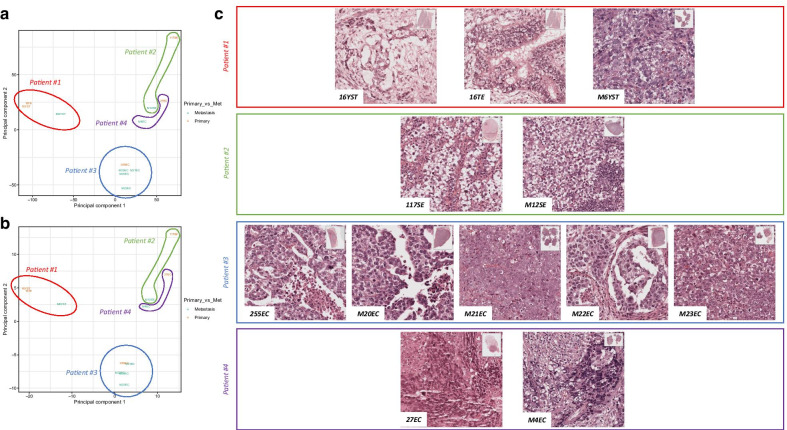
Principal component analysis of samples included in the study and histological characterization. **a**, **b** Scatter plots showing the samples' coordinates on principal components, according to individual CpG sites (**a**) and promoters (**b**). Samples are colored based on being a primary or metastatic tumor. The histological subtypes are given by the abbreviations: SE—seminoma, EC—embryonal carcinoma, YST—yolk sac tumor, TE—teratoma. Samples from the same patient were encircled together. **c** Histological representation of each sample included (×200 magnification)

We first looked at the methylation data at an exploratory level. The principal component analysis (PCA) at both CpG site (Fig. 1a) and regional (promoter, Fig. 1b) levels illustrated the heterogeneity in methylation between the various samples. This analysis evidenced that different samples from the same patient (primary and metastases) tended to aggregate together, except for the primary-metastasis pair of seminomas (117SE and M12SE). In particular, this occurred also for the five samples of patient #3, which included chemo-treated and cisplatin-resistant samples. Also, of notice, samples corresponding to most differentiated histologies (yolk sac tumor and teratoma) were mapped farther away from the other more undifferentiated subtypes. Histological representation of each sample is provided in Fig. 1c.

**Fig. 2:**
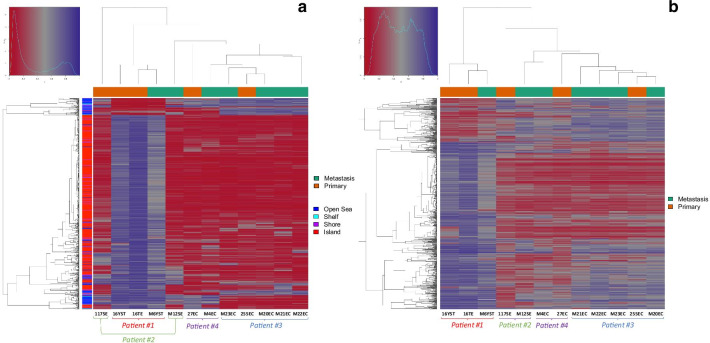
Unsupervised hierarchical clustering of samples based on all methylation values at CpG site (**a**) and promoter (**b**) level. The heatmap displays only selected sites/regions with the highest variance across all samples. Notice distinct clustering of samples from patient #1. Also, notice higher methylation density represented by higher beta values at certain CpG sites (upper right corner) and promoters (lower right corner) for patient #3 compared to patient #4 samples. For CpG site clustering, CGI relation is given on the left side of the heatmap

These data are concordant with hierarchical clustering analysis, which shows that the most differentiated tumor subtypes (YST and TE primary and metastatic samples belonging to patient #1) clustered opposite to the remaining samples, with high beta values (hypermethylation) across most individual CpG probes (Fig. 2a) and promoters (Fig. 2b). Similar to the PCA, clustering of samples per patient was observed. Particularly, embryonal carcinoma samples from patient #3 (who developed cisplatin resistance and died of disease) clustered together, and differently from embryonal carcinoma samples of patient #4 (who had a complete response to cisplatin), the former showing more evident hypermethylation of certain promoters illustrated by higher beta values.

Putting together these data, most remarkable and evident changes in methylation profile seem to be due to differences in histology between samples. However, there are also hints of differential (hyper)methylation related to response to cisplatin. To better appreciate subtle changes related to metastatic dissemination or cisplatin exposure, a differential analysis was pursued.

### Differential methylation analyses

#### Differential methylation related to histology

The number of differentially hypermethylated and hypomethylated promoters and CpG sites in each analysis explored below is found summarized in Table 2.

**Table 2:** Number of Promoters/CpG sites differentially hyper-/hypomethylated within the best 1000 combined rank

	Region level (promoters) (hyper-/hypomethylated)	Site level (CpG site) (hyper-/hypomethylated)
Histology		
NS-EC	469/38	817/183
EC-SE	467/65	794/206
YST/TE-EC	501/122	953/47
YST/TE-SE	519/31	899/101
Metastasis-primary		
Patient #1	209/324	500/500
Patient #2	321/140	405/595
Patient #3	289/210	422/578
Patient #4	337/73	655/345
Cisplatin resistant-sensitive	488/8	801/199

**Fig. 3:**
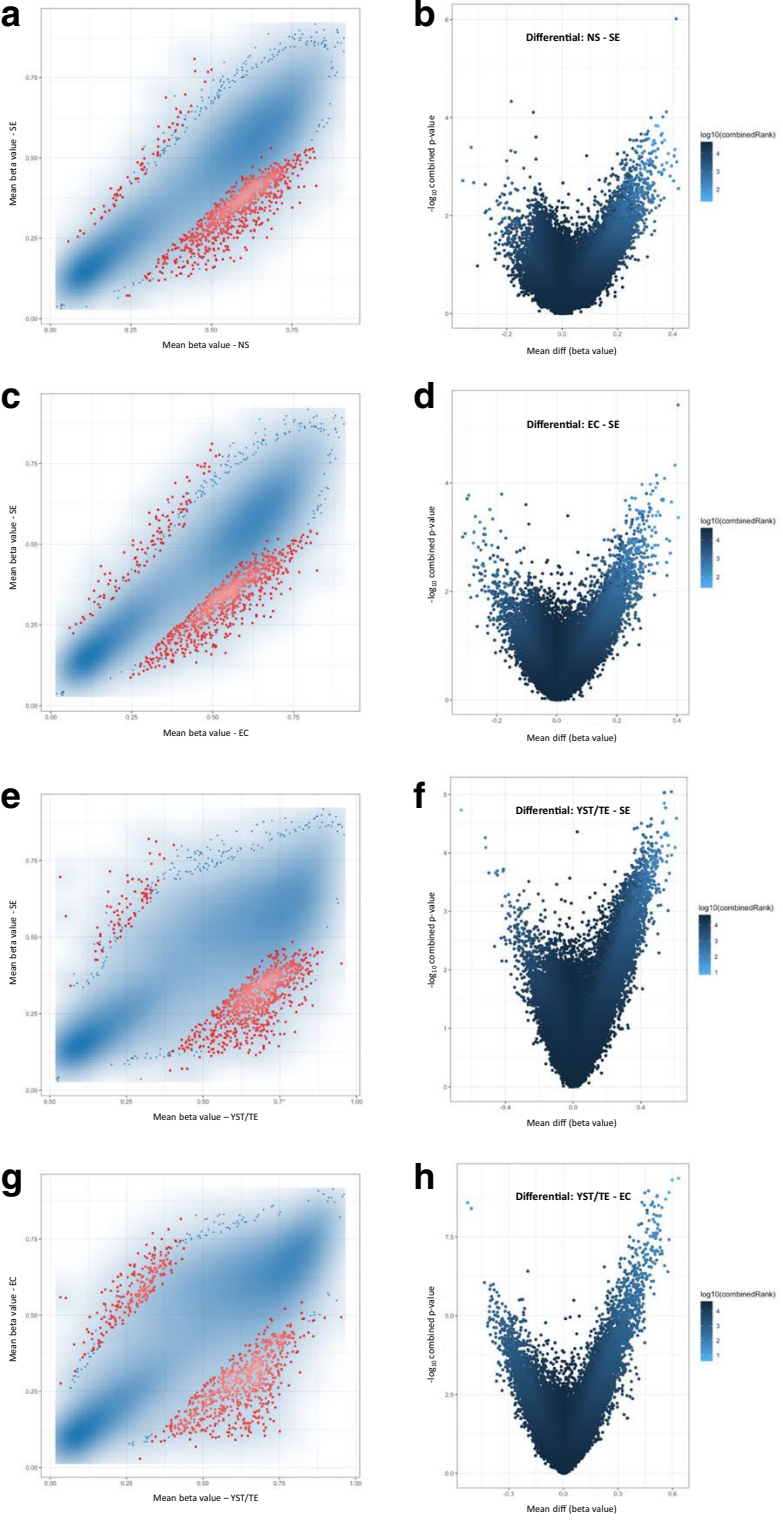
Scatterplots of the mean beta values (**a**, **c**, **e**, **g**) and volcano plots of each pairwise comparison (**b**, **d**, **f**, **h**) related to differential methylation across histologies, at the promoter level. **a**, **b** Non-seminoma–seminoma; **c**, **d** Embryonal carcinoma–seminoma; **e**, **f** Yolk sac tumor/teratoma–Seminoma; **g**, **h** Yolk sac tumor/teratoma–embryonal carcinoma. In the scatterplots, the transparency corresponds to point density. Blue points represent differentially methylated sites (according to the combined rank criteria, see Methods). Red dots represent the 1000 best ranking sites. In the volcano plots, dots are colored according to combined rank, and the *yy* axis represents the combined *p* values. *EC* embryonal carcinoma, *SE* seminoma, *TE* teratoma, *YST* yolk sac tumor

Firstly, we assessed differences in methylation profile at promoter and CpG site levels among different histologies. Figure 3 and Additional file 2: Figure S1 illustrate the differential methylation among all seminoma and non-seminoma samples, and also among individual subtypes, according to the defined criteria based on ranking. For grouping purposes, the teratoma sample was grouped with the two yolk sac tumor samples of the same patient, representing the most differentiated histologies that clustered together in previous analyses. This analysis evidenced, as expected, a differential methylation profile between non-seminoma and seminoma (with a higher density of hypermethylated promoters and sites in non-seminoma). Non-seminomas showed 469 and 817 differentially hypermethylated promoters and CpG sites, compared to only 38 and 183 hypomethylated. The same was seen for embryonal carcinoma when compared to seminoma. The most remarkable differential methylation pattern was seen when comparing yolk sac tumor/teratoma with either seminoma or embryonal carcinoma samples, the former being the samples with a higher density of hypermethylation (versus seminoma: 519 and 899 differentially hypermethylated promoters and CpG sites, and only 31 and 101 hypomethylated; versus embryonal carcinoma: 501 and 953 differentially hypermethylated promoters and CpG sites, and only 122 and 47 hypomethylated).

**Fig. 4:**
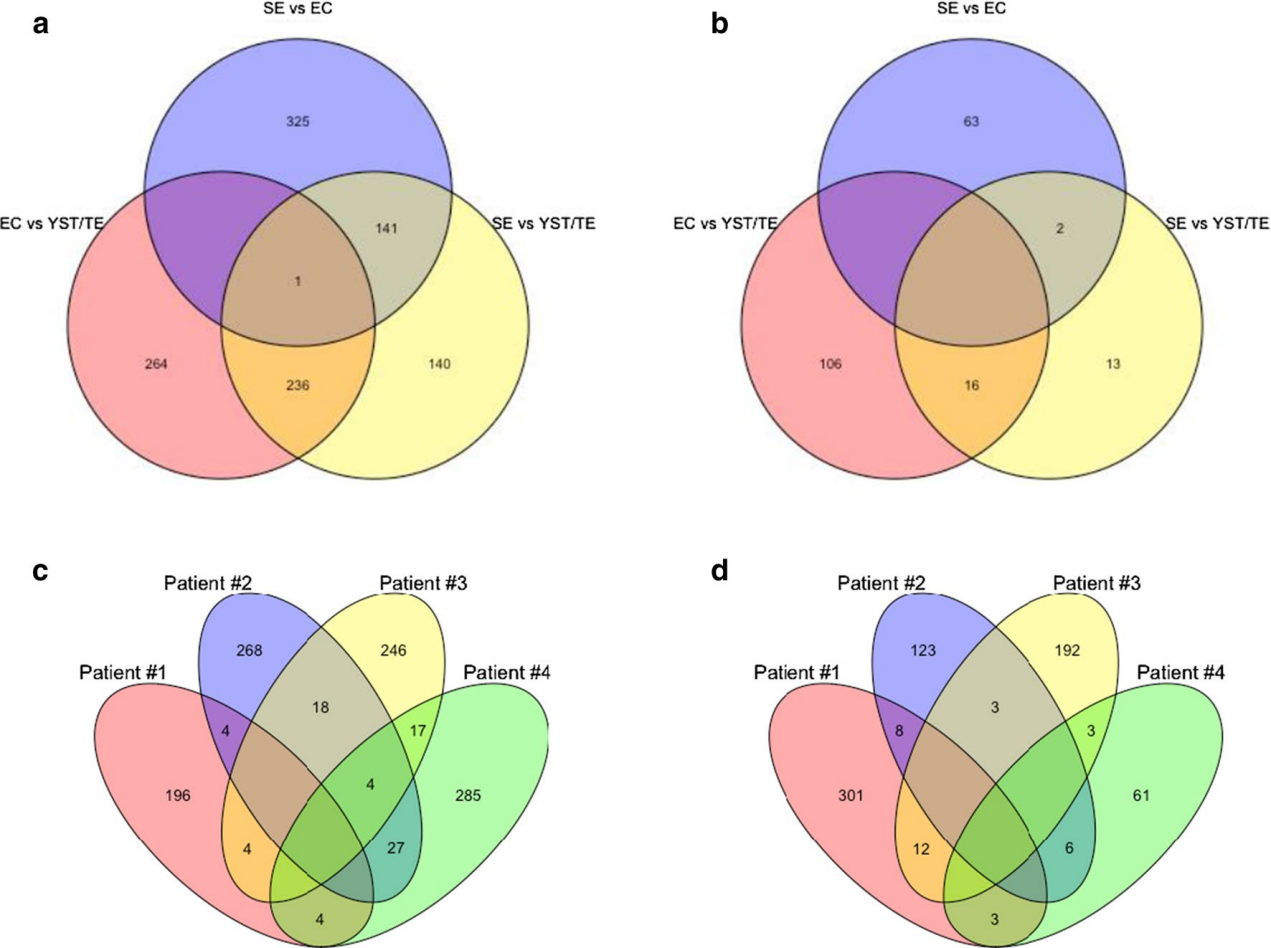
Venn diagrams analyses displaying the common and differentially hypermethylated (**a**, **c**) and hypomethylated (**b**, **d**) promoters shared by different histological subtype comparisons (**a**, **b**) and between primary-metastasis pairs belonging to different patients (**c**, **d**)

Venn diagram analyses were performed to investigate if the same differentially hypermethylated/hypomethylated promoters were shared by different histological subtype comparisons. Figure 4a, b illustrates the heterogeneity in differential methylation of specific promoters between samples of different histologies. Only one promoter (related to long non-coding RNA *ZMYND10-AS1*) was differentially hypermethylated and common to all pairwise comparisons. Most differentially hyper/hypomethylated promoters were not shared across pairwise histological comparisons (hypermethylated: 264, 325 and 140; hypomethylated: 106, 63 and 13). The histological comparisons showing most shared differentially methylated promoters were yolk sac tumor/teratoma compared to both seminoma or embryonal carcinoma (236 and 16 hypermethylated and hypomethylated promoters shared, respectively).

#### Differential methylation in primary versus metastatic samples

**Fig. 5:**
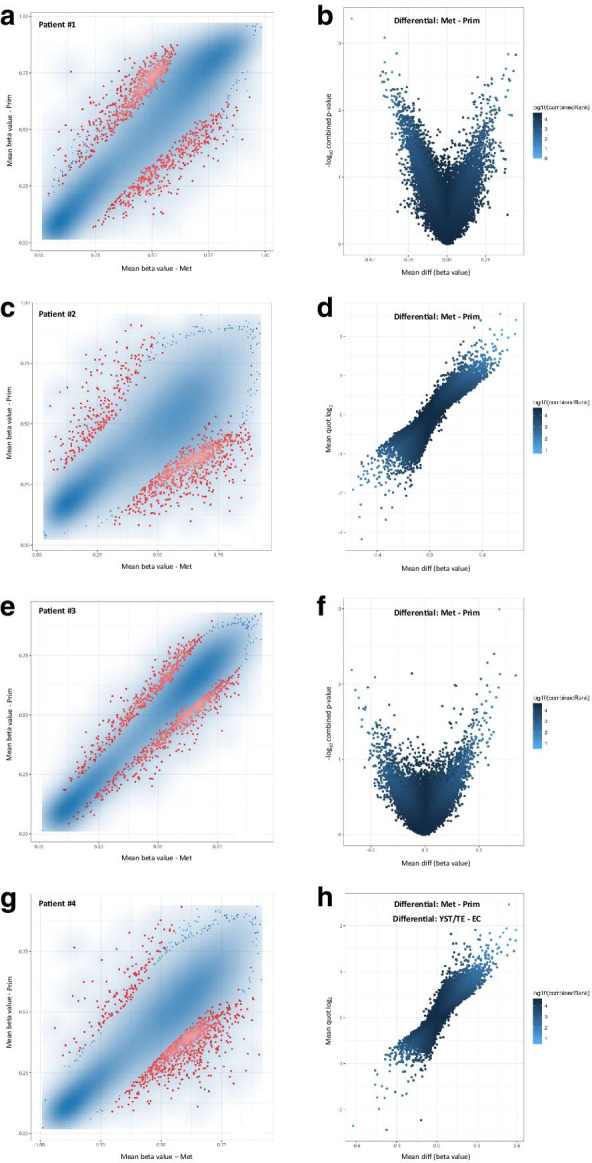
Scatterplots of the mean beta values (**a**, **c**, **e**, **g**) and volcano plots of each pairwise comparison (**b**, **d**, **f**, **h**) related to differential methylation among matched primary and metastatic samples, at the promoter level. **a**, **b**) patient #1; **c**, **d**) patient #2; **e**, **f** patient #3; **g**, **h** patient #4. In the scatterplots, the transparency corresponds to point density. Blue points represent differentially methylated sites (according to the combined rank criteria, see “Methods”). Red dots represent the 1000 best ranking sites. In the volcano plots, dots are colored according to combined rank, and the *yy* axis represents the combined *p* values (or the mean quotient log_2_ when comparing two samples). *Met* metastatic samples, *Prim* primary tumor samples

Then, we focused on differential methylation between matched primaries and metastases (paired analysis illustrated in Fig. 5, for each of the four patients). This included, for patient #3, a primary embryonal carcinoma (chemo-naïve) and four subsequent metastases in the form of cisplatin-resistance. For this patient, and additionally for patient #2 and #4, we found higher number of differentially hypermethylated promoters in the metastatic samples when compared to the corresponding primary tumors. The number of differentially hypermethylated gene promoters in patient #3 pairwise comparison was 289, while 210 were hypomethylated. At the site level, however, only patient #4 showed a higher proportion of differentially hypermethylated CpG sites in the metastatic sample.

Again, Fig. 4c, d illustrates the heterogeneity in differential methylation of specific promoters between primary-metastasis pairs belonging to different patients. The vast majority of differentially hypermethylated (Fig. 4c) and hypomethylated (Fig. 4d) promoters between matched primaries and metastases were not shared among patients (hypermethylated: 196, 268, 246 and 285 for patients #1, #2, #3 and #4, respectively; hypomethylated: 301, 123, 192 and 61 for patients #1, #2, #3 and #4, respectively).

#### Differential methylation related to clinical response to cisplatin

**Fig. 6:**
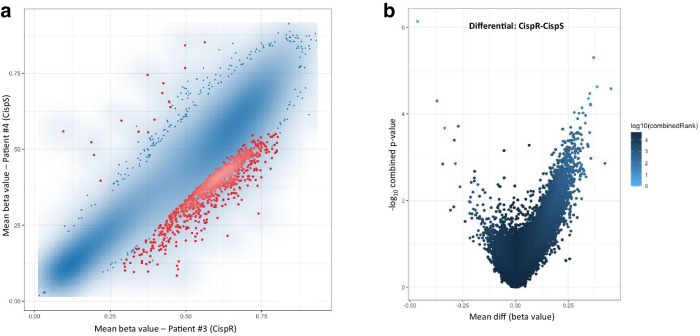
Scatterplot of the of the mean beta values (**a**) and volcano plot of the pairwise comparison (**b**) related to differential methylation among embryonal carcinoma patients with different outcome and response to cisplatin, at the promoter level. In the scatterplot, the transparency corresponds to point density. Blue points represent differentially methylated sites (according to the combined rank criteria, see Methods). Red dots represent the 1000 best ranking sites. In the volcano plot, dots are colored according to combined rank, and the *yy* axis represents the combined *p* values of a given site. *CispR* cisplatin resistant behavior, *CispS* cisplatin sensitive behavior

Finally, we investigated the differential methylation between embryonal carcinoma samples of patient #3 (cisplatin resistance emergence with viable embryonal carcinoma after chemotherapy, progression under systemic treatment ultimately dying of disease) and embryonal carcinoma samples of patient #4 (complete pathological response to cisplatin, alive with no evidence of recurrences). Since these samples have the same histological subtype both in primary and metastasis, and given the fact that samples of the same histology tended to cluster together, the paired analysis of these samples allows to look more specifically into the effect of cisplatin sensitivity/resistance (Fig. 6). We found remarkable differential methylation (both at promoter and site level, Fig. 6a, b, respectively) between these two groups of samples, with the cisplatin-resistant patient samples showing a much higher density of hypermethylation compared to cisplatin-sensitive samples (488 hypermethylated promoters compared to only 8 hypomethylated; 801 hypermethylated CpG sites compared to only 199 hypomethylated), in line with recently reported data on resistant/sensitive TGCT cell line pairs [14].

Based on this we performed GO enrichment analyses to pinpoint molecular function and pathways activated related to differentially methylated genes between cisplatin-resistant and cisplatin sensitive samples. Full list of statistically significant hits for molecular function for both hypermethylated and hypomethylated genes is provided in Additional file 1: File S1, ordered according to respective odds ratio. Illustrative figure of 100 best hits is provided in Additional file 5: Figure S4. Of notice, the differentially hypermethylated promoters in the cisplatin-resistant samples of patient #3 were involved in molecular functions related to chemokines and chemoattraction (the top three entries including CCR1, CCR6 and CCR5 chemokine receptor binding; also including “general chemokine receptor binding”, “CXCR chemokine receptor binding”, “chemoattractant activity” and “chemokine activity”). On the other hand, the differentially hypomethylated promoters in cisplatin-resistant samples were in great part enriched for functions related to nucleic acid/protein/chromatin binding and remodeling, also evidencing chromatin structure regulation as a differential aspect related to cisplatin resistance.

## Discussion

Our study confirmed that patients with distinct histologies clustered differently in regard to DNA methylation patterns, as reported [10, 11]. Samples of the same histology belonging to each individual patient clustered together in PCA (Fig. 1), irrespective of being a primary tumor or metastasis, of being chemo-naïve/exposed or of being cisplatin resistant or sensitive. This PCA is in line with the one reported by Fazal et al. [14], the first reporting EPIC array methylation analysis of cisplatin sensitive and resistant isogenic clones of TGCT cell lines. Authors also have seen major grouping of cells based on cell lineage independently of status of response to cisplatin. Moreover, our Venn diagram analysis (Fig. 4) further depicted that very few differentially hyper/hypomethylated promoters or CpG sites were shared among histology pairs or among patients, putting in evidence heterogeneity and patient-specific methylation profiles, not captured in in vitro studies. However, also like us, the authors have noticed some indications of differential clustering related to cisplatin response, and our unsupervised clustering already showed a distinct pattern of tissue samples of patient #3 (who had cisplatin resistant and poor prognosis disease) compared to the tumors (of the exact same histological subtype, embryonal carcinoma) of patient #4 (who had cisplatin sensitive disease and good outcome). This more methylated pattern was actually shared both by the embryonal carcinoma primary and respective metastases, showing that this distinctive hypermethylation was at least in part already present at diagnosis.

Cisplatin resistance is multifactorial, with several reported contributing mechanisms in TGCTs (summarized in [16, 26–30]). Recent wide studies have focused on dissecting the genomic landscape of sensitive and resistant tumors in respect to mutations and copy number changes, also showing heterogeneity [19, 22]. Very recently, Roška and collaborators very elegantly identified putative mRNA-based biomarkers of cisplatin resistance by making use of sensitive and resistant cell lines [23]. Data on DNA methylation is less studied [17, 31]. Hypermethylation of selected gene promoters, such as *CALCA*, *MGMT* and *RASSF1A*, have been associated with resistant phenotype in few individual studies [31, 32]. A broader understanding of these mechanisms is key for uncovering novel clinically useful biomarkers and targeted therapies for these patients [33]. The heterogeneity found between individual differentially methylated promoters and CpG sites in our proof-of-concept, discovery work indicates that finding a universal methylation-based biomarker to predict resistance may be difficult. Larger studies on patients sharing the same histology and characteristics will be instrumental to answer this question and can only be achieved by multi-institutional international cooperation, with collection of tissue samples from truly cisplatin resistant metastatic disease.

The mentioned cell line study by Fazal et al. [14] showed a net increase in overall methylation related to acquisition of the resistant phenotype. Authors have used a methodological approach similar to ours (although with some difference in the consideration of differentially methylated samples, using a beta value > 0.2 and FDR < 0.05, while we computed a combined rank). Our differential analysis of embryonal carcinoma samples from patients with distinct response to cisplatin disclosed this same increase at tissue level, validating the in vitro results. Both proof-of-concept studies constitute an argument in favor of using hypomethylating targeted treatments for these resistant patients, which was one of the main research questions investigated in our discovery work. In fact, the same group and others have already shown the benefit of agents such as 5-AZA, DAC and guadecitabine in treating resistant patients and in rescuing sensitivity to cisplatin, both in vitro and including in a recent clinical trial [34–38].

Finally, our study revealed among the top differentially hypermethylated promoters related to cisplatin resistance targets involved in pathways related to chemoattraction and hence immune infiltration of the tumor bed (Additional file 1: File S1). Studies of immunotherapies in heavily treated and refractory TGCT patients have not produced the most ideal clinical benefit [39–41]. The use of demethylating agents (or of HDAC inhibitors) could produce epigenetic priming of the tumor, turning it into a “hot tumor”, more responsive to anti-PD1/PDL1 therapies [42]. Our data further strengthens this combination [43], which could be explored in future studies in TGCTs. Moreover, the differentially hypomethylated promoters in the cisplatin resistant context were enriched in molecular functions related to DNA binding and chromatin remodeling. This is also in line with results of Fazal et al. [14] and further studies of this group [15], indicating that therapies aiming at targeting chromatin remodelers can also be envisioned to target cisplatin resistant tumors, as suggested [44, 45].

One limitation of our work is related to the small amount of samples included in the study (*n* = 12). However, as mentioned, having tissue samples of primary tumor and matched metastasis (which are infrequently sampled) with sufficient amount of tumor cells for performing EPIC array is rare, hence the scarcity of studies with this matched primary-metastasis framework. Also, we have access to detailed clinical information about these patients, which enriched our conclusions and analysis. Furthermore, importantly, we made use of a strict combined rank for analyzing the differential methylation data, which took into account both p-values and fold-change of difference and quotient between beta values, further increasing the robustness of the findings. Also, presently we cannot, further correlate DNA methylation data with specific gene expression, as no more tumor material is left in tumor blocks of some metastatic samples, deriving from small needle biopsies / small resections. We are currently prospectively collecting further tissue samples, which are totally embedded, in our Institute, but think that such studies will be for sure facilitated by multicentric international collaboration. Additionally, publicly available databases including also metastatic samples from these patients are not available or well characterized, which further difficult such studies.

Our results in patient samples confirm histology as being a major determinant of methylation profile (validating previous data now with the EPIC 850 k array) and also validate the recent report obtained in vitro, further confirming its methodological robustness. Our aim was not to provide an investigation and validation of specific methylation biomarkers of cisplatin resistance (which, based on our and others’ data showing tremendous heterogeneity, may be actually quite challenging in tissue studies). Instead, we provide a proof-of-concept, discovery, setting on patient-derived samples for supporting that increased overall methylation associates with cisplatin resistant phenotype. Importantly, we believe our study will further encourage investigation on hypomethylating drug compounds for treating these patients, strengthening this particular field of research.

## Supplementary Information

**Additional file 1: File S1.** GO enrichment analysis, depicting the molecular functions of differentially hypermethylated and hypomethylated promoters among embryonal carcinoma samples belonging to a cisplatin resistant patient with poor clinical outcome and a cisplatin sensitive patient with good clinical outcome. Only significant (p<0.05) entries are displayed, ordered by decreasing odds ratio.

**Additional file 2: Figure S1.** Scatterplots of the mean beta values (A, C, E, G) and volcano plots of each pairwise comparison (B, D, F, H) related to differential methylation across histologies, at the CpG site level. A-B) Non-seminoma - Seminoma; C-D) Embryonal carcinoma - Seminoma; E-F) Yolk sac tumor/teratoma - Seminoma; G-H) Yolk sac tumor/teratoma – Embryonal carcinoma. In the scatterplots, the transparency corresponds to point density. Blue points represent differentially methylated sites (according to the combined rank criteria, see Methods). Red dots represent the 1000 best ranking sites. In the volcano plots, dots are colored according to combined rank, and the yy axis represents the combined p-values. Abbreviations: EC – embryonal carcinoma; SE – seminoma; TE – teratoma; YST – yolk sac tumor.

**Additional file 3: Figure S2.** Scatterplots of the mean beta values (A, C, E, G) and volcano plots of each pairwise comparison (B, D, F, H) related to differential methylation among matched primary and metastatic samples, at the CpG site level. A-B) patient #1; C-D) patient #2; E-F) patient #3; G-H) patient #4. In the scatterplots, the transparency corresponds to point density. Blue points represent differentially methylated sites (according to the combined rank criteria, see Methods). Red dots represent the 1000 best ranking sites. In the volcano plots, dots are colored according to combined rank, and the yy axis represents the combined p-values (or the mean quotient log2 when comparing two samples). Abbreviations: Met – metastatic samples; Prim – primary tumor samples.

**Additional file 4: Figure S3.** Scatterplot of the of the mean beta values (A) and volcano plot of the pairwise comparison (B) related to differential methylation among embryonal carcinoma patients with different outcome and response to cisplatin, at the CpG site level. In the scatterplot, the transparency corresponds to point density. Blue points represent differentially methylated sites (according to the combined rank criteria, see Methods). Red dots represent the 1000 best ranking sites. In the volcano plot, dots are colored according to combined rank, and the yy axis represents the combined p-values of a given site. Abbreviations: CispR – cisplatin resistant behavior; CispS – cisplatin sensitive behavior.

**Additional file 5: Figure S4.** Molecular functions related to differentially hypermethylated (A) and hypomethylated (B) promoters. Statistically significant and top hits (odds ratio) are illustrated.

## Data Availability

Data generated or analyzed during this study are included in this article and its supplementary information files.
