# Supplementary figures and images for: Differential methylation EPIC analysis discloses cisplatin-resistance related hypermethylation and tumor-specific heterogeneity within matched primary and metastatic testicular germ cell tumor patient tissue samples
Source: Clin Epigenetics. 2021 Apr 6;13:70. doi: 10.1186/s13148-021-01048-y (PMC8025580; doi:10.1186/s13148-021-01048-y)

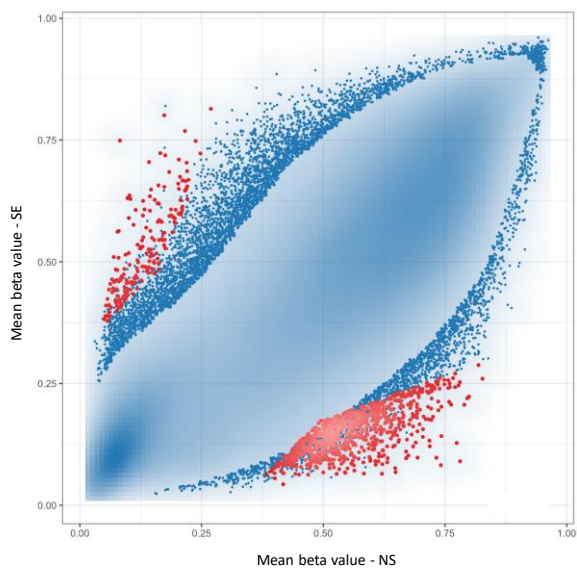

**A**

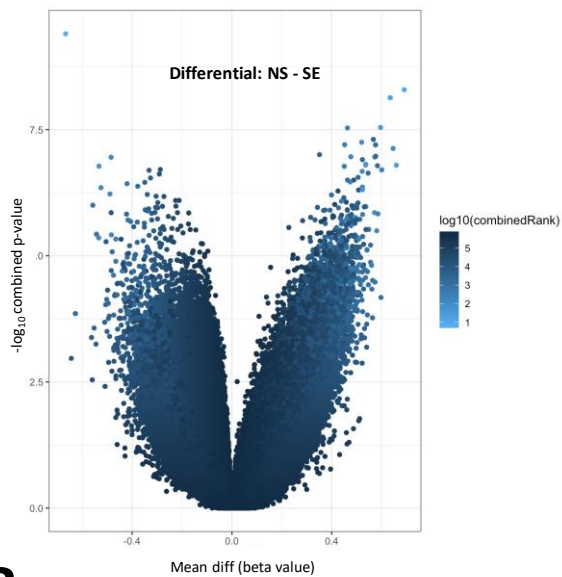

**B**

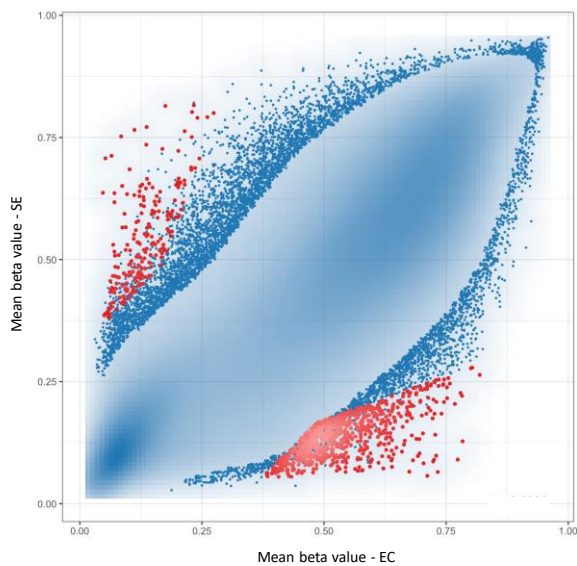

**C**

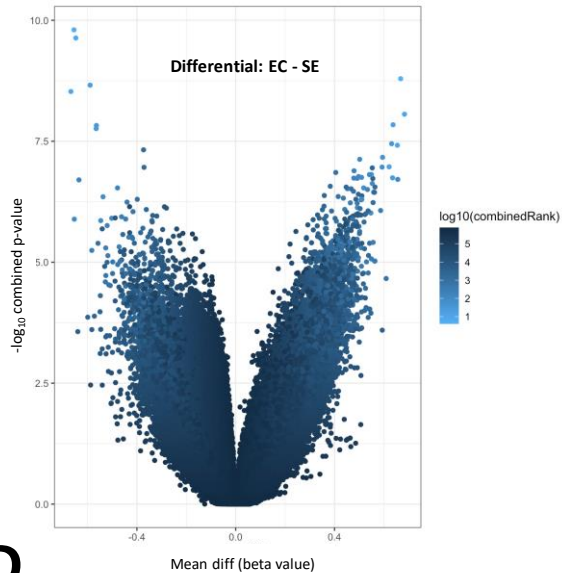

**D**

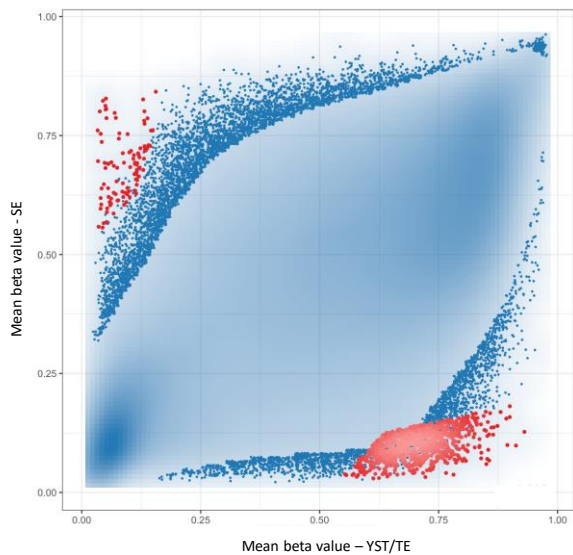

**E**

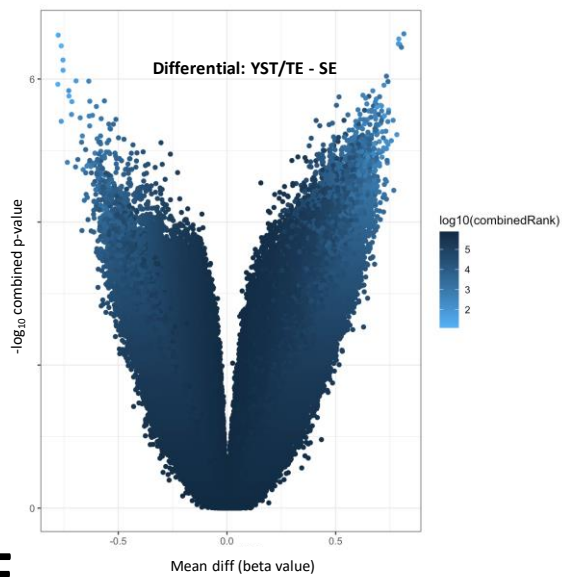

**F**

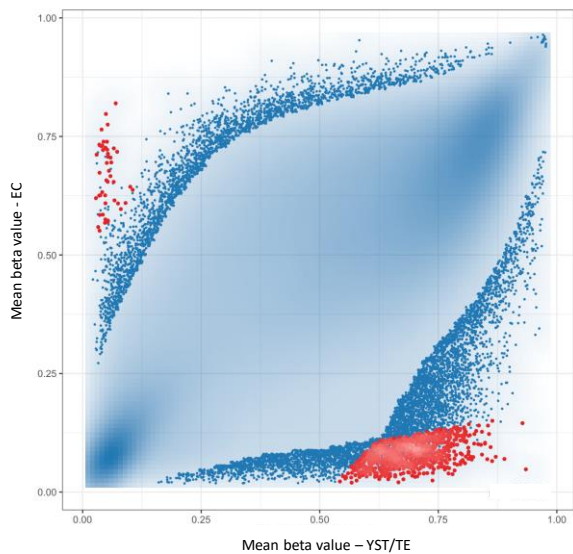

**G**

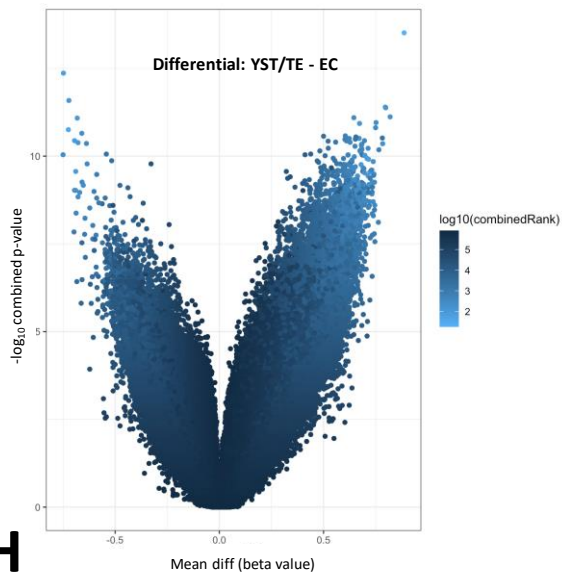

**H**

Supplement: Supplementary file 2 — Additional file 2: Figure S1. Scatterplots of the mean beta values (A, C, E, G) and volcano plots of each pairwise comparison (B, D, F, H) related to differential methylation across histologies, at the CpG site level. A-B) Non-seminoma - Seminoma; C-D) Embryonal carcinoma - Seminoma; E-F) Yolk sac tumor/teratoma - Seminoma; G-H) Yolk sac tumor/teratoma – Embryonal carcinoma. In the scatterplots, the transparency corresponds to point density. Blue points represent differentially methylated sites (according to the combined rank criteria, see Methods). Red dots represent the 1000 best ranking sites. In the volcano plots, dots are colored according to combined rank, and the yy axis represents the combined p-values. Abbreviations: EC – embryonal carcinoma; SE – seminoma; TE – teratoma; YST – yolk sac tumor. [file 13148_2021_1048_MOESM2_ESM.pdf]

**A**

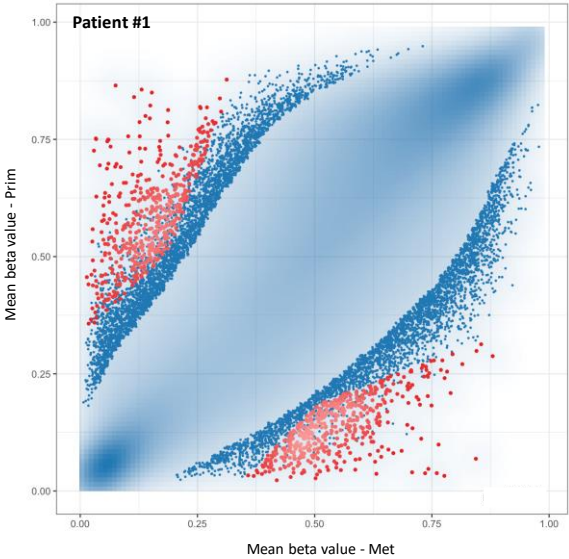

**B**

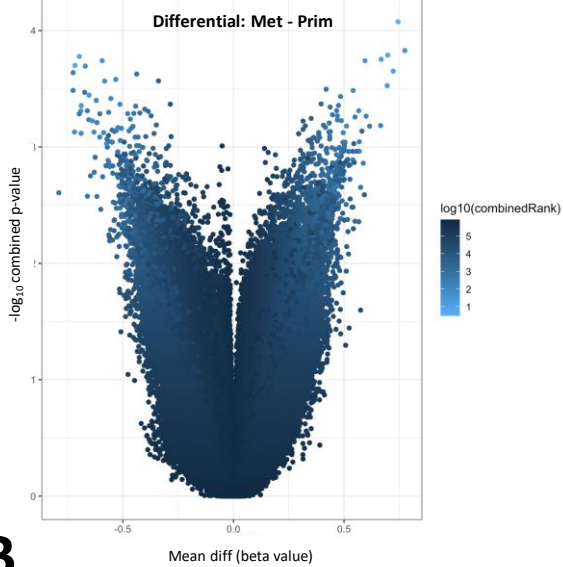

**C**

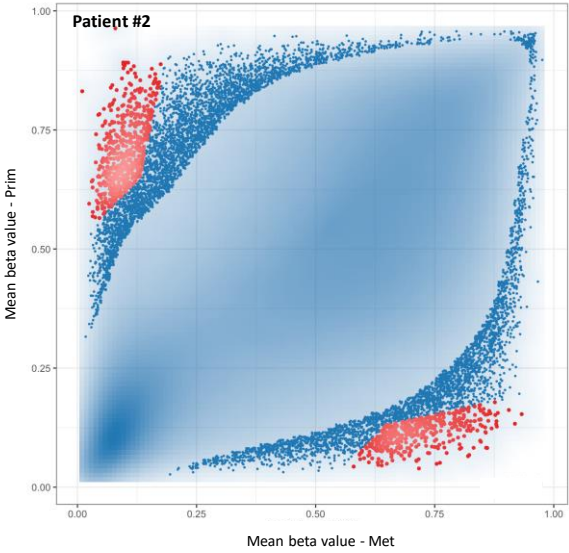

**D**

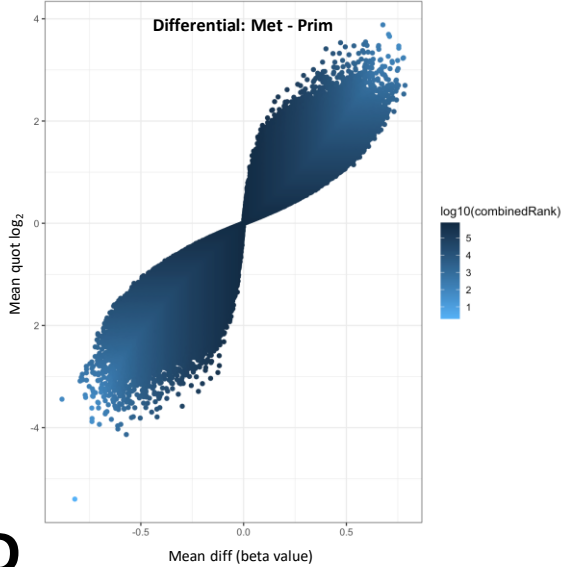

**E**

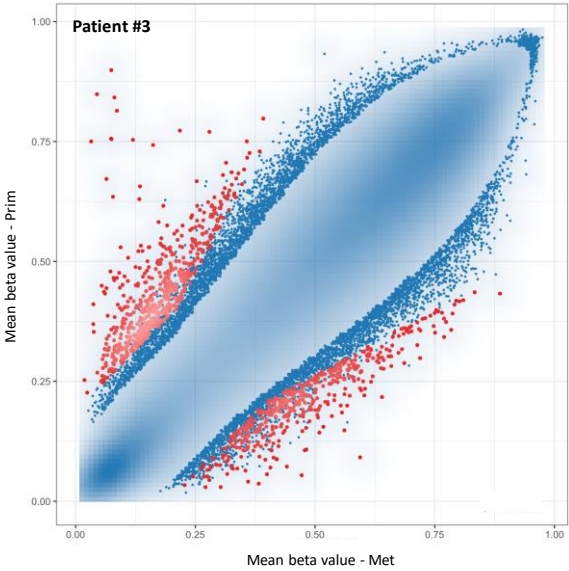

**F**

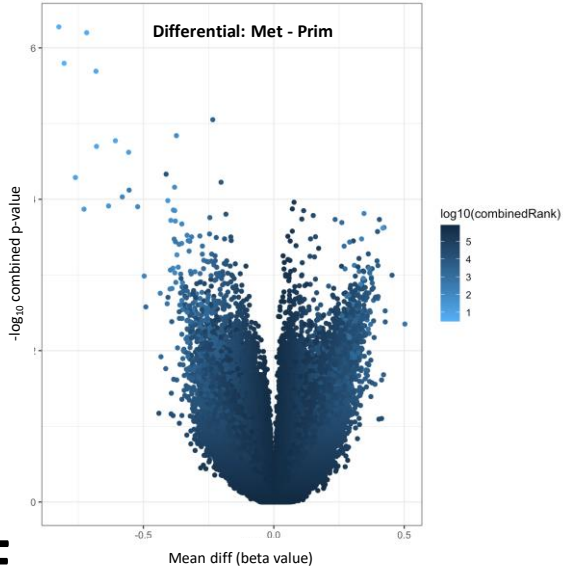

**G**

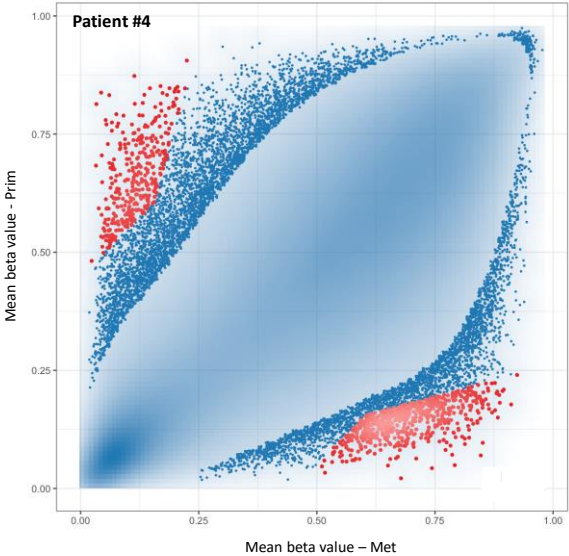

**H**

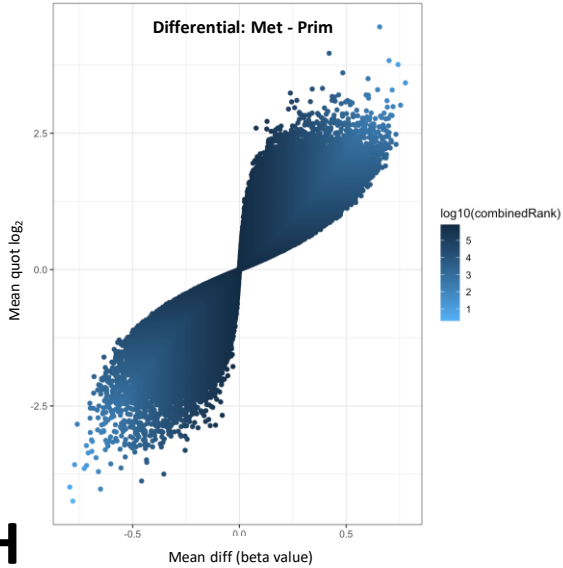

Supplement: Supplementary file 3 — Additional file 3: Figure S2. Scatterplots of the mean beta values (A, C, E, G) and volcano plots of each pairwise comparison (B, D, F, H) related to differential methylation among matched primary and metastatic samples, at the CpG site level. A-B) patient #1; C-D) patient #2; E-F) patient #3; G-H) patient #4. In the scatterplots, the transparency corresponds to point density. Blue points represent differentially methylated sites (according to the combined rank criteria, see Methods). Red dots represent the 1000 best ranking sites. In the volcano plots, dots are colored according to combined rank, and the yy axis represents the combined p-values (or the mean quotient log2 when comparing two samples). Abbreviations: Met – metastatic samples; Prim – primary tumor samples. [file 13148_2021_1048_MOESM3_ESM.pdf]

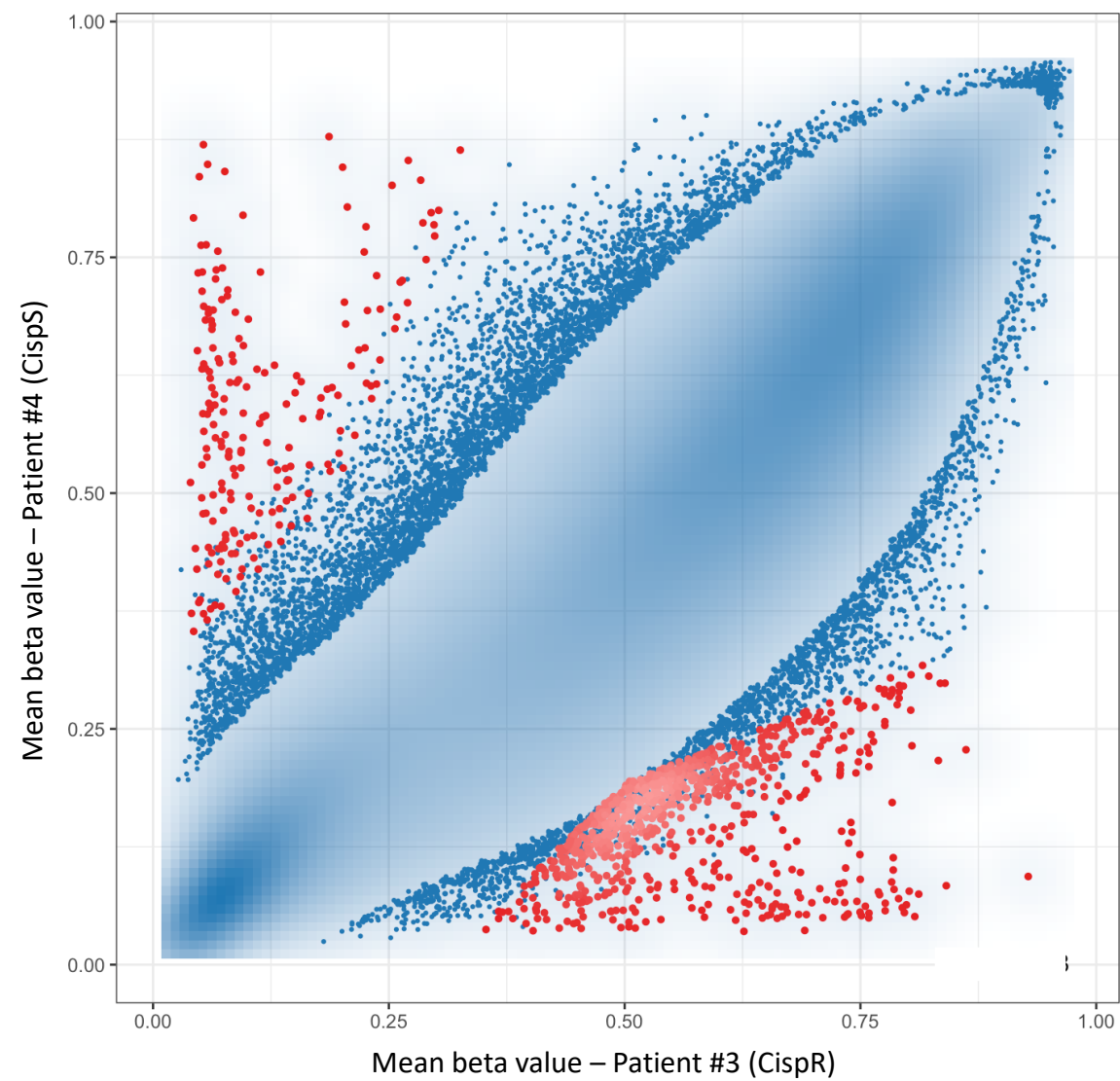

**A**

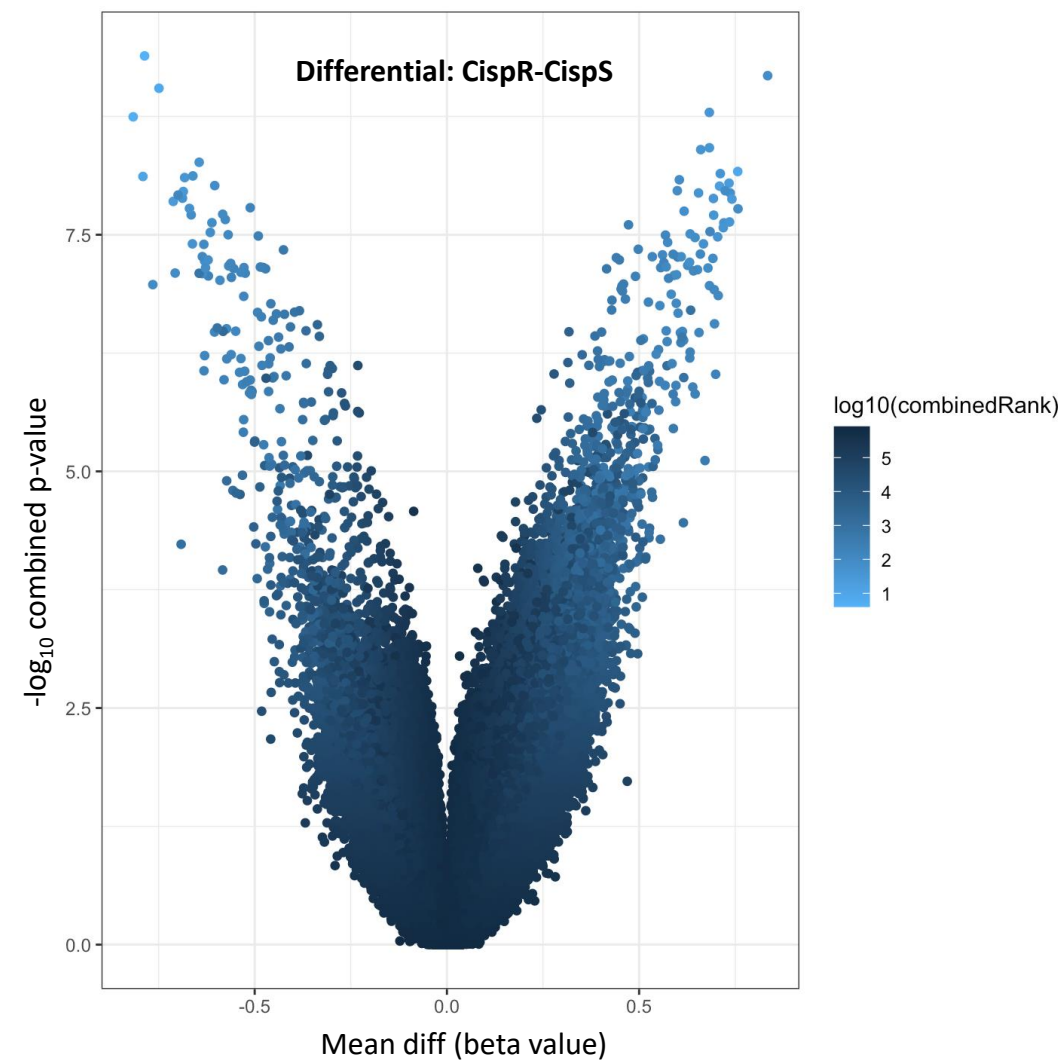

**B**

Supplement: Supplementary file 4 — Additional file 4: Figure S3. Scatterplot of the of the mean beta values (A) and volcano plot of the pairwise comparison (B) related to differential methylation among embryonal carcinoma patients with different outcome and response to cisplatin, at the CpG site level. In the scatterplot, the transparency corresponds to point density. Blue points represent differentially methylated sites (according to the combined rank criteria, see Methods). Red dots represent the 1000 best ranking sites. In the volcano plot, dots are colored according to combined rank, and the yy axis represents the combined p-values of a given site. Abbreviations: CispR – cisplatin resistant behavior; CispS – cisplatin sensitive behavior. [file 13148_2021_1048_MOESM4_ESM.pdf]
