# Supplementary material for: Differential methylation EPIC analysis discloses cisplatin-resistance related hypermethylation and tumor-specific heterogeneity within matched primary and metastatic testicular germ cell tumor patient tissue samples
Source: Clin Epigenetics. 2021 Apr 6;13:70. doi: 10.1186/s13148-021-01048-y (PMC8025580; doi:10.1186/s13148-021-01048-y)
Supplement: Supplementary file 5 — Additional file 5: Figure S4. Molecular functions related to differentially hypermethylated (A) and hypomethylated (B) promoters. Statistically significant and top hits (odds ratio) are illustrated. [file 13148_2021_1048_MOESM5_ESM.pdf]

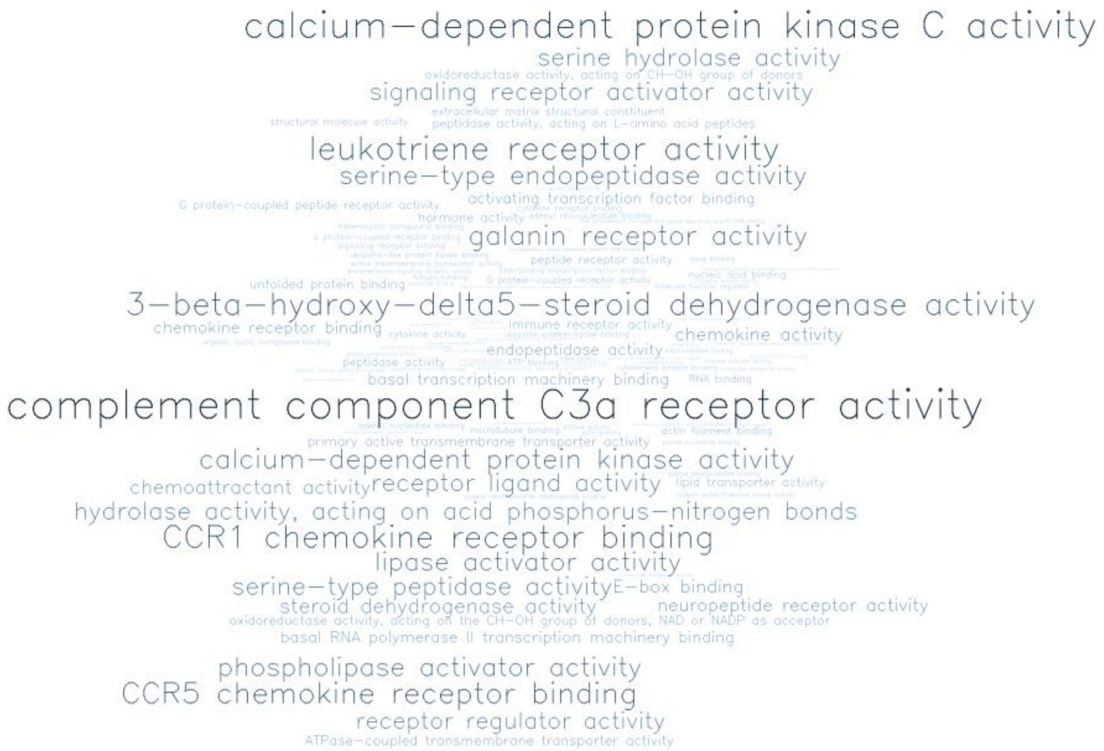

calcium−dependent protein kinase C activity
serine hydrolase activity
oxidoreductase activity, acting on CH−OH group of donors
signaling receptor activator activity
extracellular matrix structural constituent
structural molecule activity
peptidase activity, acting on L−amino acid peptides
leukotriene receptor activity
serine−type endopeptidase activity
G protein−coupled peptide receptor activity
activating transcription factor binding
hormone activity
galanin receptor activity
peptide receptor activity
unfolded protein binding
G protein−coupled receptor activity
nucleic acid binding
3−beta−hydroxy−delta5−steroid dehydrogenase activity
chemokine receptor binding
immune receptor activity
chemokine activity
cytokine activity
endopeptidase activity
peptidase activity
ATP binding
basal transcription machinery binding
RNA binding
complement component C3a receptor activity
microtubule binding
actin filament binding
primary active transmembrane transporter activity
calcium−dependent protein kinase activity
chemoattractant activity
receptor ligand activity
lipid transporter activity
hydrolase activity, acting on acid phosphorus−nitrogen bonds
CCR1 chemokine receptor binding
lipase activator activity
serine−type peptidase activity
E−box binding
steroid dehydrogenase activity
neuropeptide receptor activity
oxidoreductase activity, acting on the CH−OH group of donors, NAD or NADP as acceptor
basal RNA polymerase II transcription machinery binding
phospholipase activator activity
CCR5 chemokine receptor binding
receptor regulator activity
ATPase−coupled transmembrane transporter activity

A

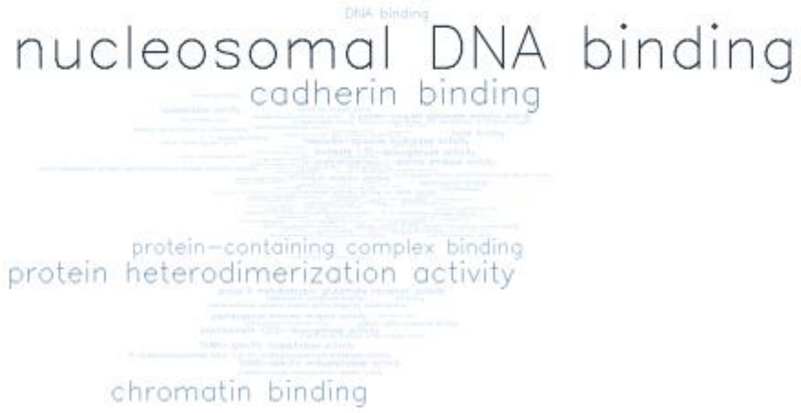

DNA binding
nucleosomal DNA binding
cadherin binding
protein−containing complex binding
protein heterodimerization activity
chromatin binding

B
